# Chromosome-level genome assembly of the cereal cyst nematode *Heterodera flipjevi*

**DOI:** 10.1038/s41597-024-03487-7

**Published:** 2024-06-17

**Authors:** Ke Yao, Jiangkuan Cui, Jinzhuo Jian, Deliang Peng, Wenkun Huang, Lingan Kong, Qianghui Wang, Huan Peng

**Affiliations:** 1 State Key Laboratory for Biology of Plant Diseases and Insect Pests, Institute of Plant Protection, Chinese Academy of Agricultural Sciences, Beijing, 100193 China; 2 National Key Laboratory of Wheat and Maize Crop Science, College of Plant Protection, Henan Agricultural University, Zhengzhou, 450002 China; 3 Zhongyuan Research Center, Chinese Academy of Agricultural Sciences, Xinxiang, 453000 China; 4 Novogene, Bioinformatics Institute, Beijing, 100193 China

**Keywords:** Genome assembly algorithms, Genome-wide association studies, Gene ontology, Pathogens

## Abstract

As an economically important plant parasitic nematode (PPN), *Heterodera filipjevi* causes great damage on wheat, and now it was widely recorded in many countries. While multiple genomes of PPNs have been published, high-quality genome assembly and annotation on *H. filipjevi* have yet to be performed. This study presents a chromosome-scale genome assembly and annotation for *H. filipjevi*, utilizing a combination of Illumina short-read, PacBio long-read, and Hi-C sequencing technologies. The genome consists of 9 pseudo-chromosomes that contain 134.19 Mb of sequence, with a scaffold N50 length of 11.88 Mb. In total, 10,036 genes were annotated, representing 75.20% of the total predicted protein-coding genes. Our study provides the first chromosome-scale genome for *H. filipjevi*, which is also the inaugural high-quality genome of cereal cyst nematodes (CCNs). It provides a valuable genomic resource for further biological research and pest management of cereal cyst nematodes disease.

## Background & Summary

The cereal cyst nematodes (CCNs) are a group of 12 closely related species and considered to be one of the most damaging plant parasitic nematodes (PPNs) that limit production of cereal crops in many parts of the world including Australia, China, India, USA, Europe, North Africa and West Asia^1,2^. The species *Heterodera filipjevi, H. avenae*, and *H. litipones* are among the most economically important species and caused significant economic losses^3^. The yield losses caused by CCNs have been recorded from 10–35% on wheat in China, 24% on spring wheat in Oregon, and 20% on barley in Australia^4^. Among the CCNs, *H. filipjevi* is an important constraint to cereal crop production in different climatic regions^1^, and now it was widely recorded in many countries such as Tadzhikistan, Russia, Morocco, Tunisia, Pakistan, Libya, Turkey^5,6^, Estonia, Sweden, India, Norway, Iran, China^7^, United Kingdom, and USA^8^. Smiley *et al*.^4^ reported a 35% yield loss in spring wheat in Oregon, USA, due to *H. filipjevi*^4^, and Karimipour *et al*.^9^ estimated yield losses in wheat yield ranging between 20% and 25% in Iran by the same nematode species^9^. Also, Hajihasani *et al*.^10^ reported that grain yield loss caused by *H. filipjevi* occurred even at the lowest population density and reached a maximum loss of 48% with an initial population density (Pi) of 20 eggs and J2/ (g soil)^–1^ in Iran^10^.

Genomic data have proven to be powerful tools to explain the successful parasitization of plant nematodes. The first plant-parasitic nematodes genomes *Meloidogyne incognita* and *M. hapla* have been published in 2008. Recently, several PPNs genomes from *Globodera pallida*^11^, *Globodera rostochiensis*^12^, *Heterodera glycines*^13,14^, *Bursaphelenchus xylophilus*^15^, *Bursaphelenchus mucronatus*^16^, *Ditylenchus destructor*^17^, *M. floridensis*^18^ and *M. graminicola*^19^ have been published. However, the available reference genome of CCNs was absent, only the transcriptome of *H. avenae* was sequenced using short-read sequencing technology^20–23^. In the present study, a total of 95.79 Gb (711.56 x) raw data was obtained by Illumina, PacBio, 10x Genomics and Hi-C technologies, the detailed sequencing data were summarized in Table 1. The 17-mers were counted as 17,119,184,513 from 21.8 G Illumina short reads, and the k-mer depth was 124 (Table 1). Then, we used PacBio long-read, Illumina short-read, 10 × Genomics and Hi-C data to generate a high-quality chromosome-level reference genome for *H.filipjevi*. The genome assembly spanned 134.19 Mb with a scaffold N50 length of 11.88 Mb (Table 2). After chromosome-level anchoring, 9 chromosomes with a total length of 120 Mb (89.4% of the draft assembly) were constructed corresponding to the karyotype. In addition, the mapping rate of Illumina short reads was 93.14% and the genome coverage was 99.71% (Table 3). Moreover, 2,226 homozygous single-nucleotide polymorphisms (SNP) and a low homozygous rate (0.0032%) were identified, suggesting a low error rate in the assembly (Table 4). In conclusion, our evaluation indicated a high quality of the assembled *H. filipjevi* genome. Finally, we annotated 13,352 protein-coding genes in *H. filipjevi* genome with a mean of 8.14 exons per gene (Table 5 and Table 6) and found 61.9 Mb (46.14%) repeat elements. The reference genome obtained in this study will provide a foundation for future investigations on the pathogenesis of CCNs.

**Table 1:** Sequencing data used for the genome *Heterodera flipjevi* assembly.

Llibrary types	Insert size	Total data (Gb)	Sequence coverage (X)
Illumina reads	350 bp	21.80	161.94
PacBio reads	12–20 kb	14.74	109.49
10x Genomics	—	42.58	316.30
Hi-C reads	350 bp	16.67	123.83
Total	—	95.79	711.56

**Table 2:** The statistics of length and number for the *de novo* assembled *Heterodera flipjevi* genome.

Term	Length		No.	
	Contigs (bp)	Scaffolds (bp)	Contigs	Scaffolds
Total	133,432,049	134,189,547	1,208	661
Max	3,759,560	30,258,874	—	—
Number > = 2000	—	—	1,208	661
N50	445,933	11,883,475	88	4
N60	330,012	11,792,251	123	5
N70	226,932	9,490,179	173	7
N80	101,494	8,670,217	261	8
N90	40,172	83,131	474	17

**Table 3:** Statistics of reads coverage of the *Heterodera flipjevi* genome.

		Percentage (%)
Reads	Mapping rate (%)	93.14
Genome	Average sequencing depth	99.71
	Coverage (%)	95.38
	Coverage at least 4x (%)	92.28
	Coverage at least 10x (%)	84.97

**Table 4:** SNP statistics of the *Heterodera flipjevi* genome.

	Number	Percentage (%)
All SNP	384,284	0.5588
Heterozygosis SNP	382,058	0.5556
Homology SNP	2,226	0.0032

**Table 5:** Statistical results of the repetitive sequences of the *Heterodera flipjevi* genome.

Type	Repeat size (bp)	Percentage of genome (%)
Trf	14,118,665	10.52
Repeatmasker	57,239,692	42.66
Proteinmask	5,409,315	4.03
Total	61,912,430	46.14

**Table 6:** Gene annotation of *Heterodera flipjevi* genome via three methods.

Method	Gene set	Number	Average length (bp)				Exons No. Per gene
			Transcript	CDS	Exon	Intron	
*De novo*	Augustus	11,629	3,192.26	1,271.77	153.71	264.03	8.27
	GlimmerHMM	25,748	2,272.08	765.28	163.15	408.28	4.69
	SNAP	18,364	3,813.03	841.70	126.77	526.89	6.64
	Geneid	5,619	15,312.76	841.29	143.91	2,986.24	5.85
	Genscan	9,440	8,573.53	1,387.77	206.69	1,257.53	6.71
Homolog	*B. malayi*	4,902	2,214.49	1,044.08	163.53	217.36	6.38
	*C. elegans*	5,096	2,117.53	1,043.44	170.43	209.68	6.12
	*D. melanogaster*	2,469	1,808.99	906.24	163.33	198.48	5.55
	*G. pallida*	11,253	2,358.22	1,071.09	181.85	263.23	5.89
	*G. rostochiensis*	12,901	2,477.20	1,169.09	180.15	238.30	6.49
	*H. contortus*	5,677	2,164.58	1,105.42	188.18	217.30	5.87
	*H. glycines*	13,160	2,497.24	1,212.29	183.66	229.43	6.60
	*H. sapiens*	2,822	1,721.99	890.44	167.03	192.00	5.33
	*M. hapla*	9,276	1,973.10	1,036.92	193.31	214.52	5.36
	*M. incognita*	9,919	2,087.29	1,067.24	187.72	217.71	5.69
	*P. pacificus*	4,241	2,119.06	1,064.59	187.45	225.35	5.68
RNAseq	PASA	58,757	2,452.79	951.93	151.17	283.35	6.30
	Cufflinks	25,678	7,001.39	3,024.07	336.44	497.88	8.99
Final		13,352	3,258.25	1,235.80	151.89	283.41	8.14

Note that CDS refers to coding sequence; GlimmerHMM was a new genefinder based on a Generalized Hidden Markov Model (GHMM); SNAP refers to Semi-HMM-based Nucleic Acid Parser; EVM refers to Evidence modeler.

## Methods

### Nematode sample and DNA extraction

The cysts of *H. filipjevi* were collected from wheat fields in Xuchang city Henan province. Ten cysts were chosen and inoculated on susceptible wheat cultivars Wenmai 19 in greenhouse for 6 generations. The fresh, healthy and unbroken cysts were manually picked and used for extraction of eggs by crushed in sterile water. the eggs were subsequently collected using the sucrose flotation technique^24^ and cleaned with sterile distilled water for three times. Six developmental stages of *H. flipjevi* including pre-parasitic second stage juveniles (Pre-J2), parasitic-J2 (Para-J2), third stage juveniles (J3), fourth stage juveniles 4 (J4), adult females (Fes) and eggs were collected according to the previous report^20^.

### DNA isolation and sequencing

Genomic DNA was isolated from *H. filipjevi* egg mass according to the CTAB method. The DNA quality and concentration were assessed using agarose gel electrophoresis and a Qubit 2.0 Fluorometer (Life Technologies, CA, USA). A 20 kb insert sizes library was produced following the manufacturer’s protocol (PacBio, CA) and sequenced with the PacBio RS technology. For short-read sequencing, libraries with 350 bp insert sizes was prepared and sequenced on Illumina HiSeq 2500 as 2 × 150 bp reads (Table 1). The GEM (Gel Beads in Emulsion) reaction was conducted for 10 × Genomics sequencing using approximately 1 ng input DNA of 50 kb length, and 16 barcodes were introduced into droplets, subsequence, the droplets were fractured and 600 bp fragments were used for constructing libraries, which were sequenced on the Illumina HiSeq X platform at the Novogene Bioinformatics Institute, Beijing.

### Genome size estimation, assembly and evaluation

For survey analysis, the *H. filipjevi* genome size was estimated using the 21.8 Gb paired-end Illumina sequencing data, based on the K-mer formula: Genome size = (total number of 17-mer) / (position of the homozygous peak). With the 14.74 Gb long reads generated from PacBio Sequel platform, the contig assembly of H. filipjevi genome was conducted using the FALCON assembler (version 1.2.4)^25^. Then, the assembly from PacBio data was polished by Quiver (smrtlink 5.0.1)^26^. The heterozygosity of assembly was removed by using the Purge Haplotigs software (version 1.1.1)^27^. The resulting contigs were connected to super-scaffolds by 42.58 Gb 10 × Genomics linked-read data using the fragScaff software (Version 140324)^28^ (Table 1). Finally, the short reads from Illumina were used to correct any remaining errors by Pilon (Version 1.22)^29^. These processes would yield a final draft *H. filipjevi* genome.

**Fig. 1:**
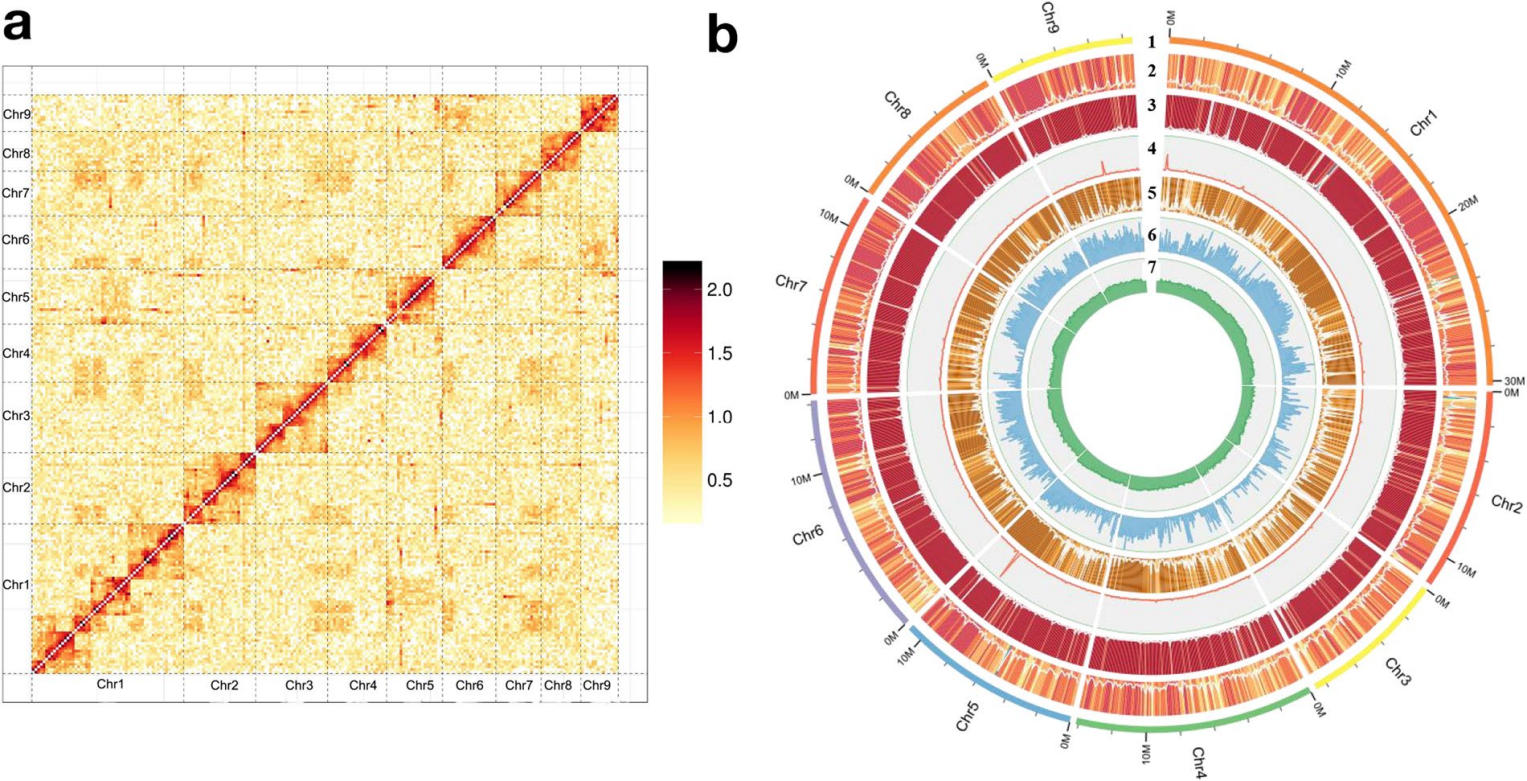
Characteristics of the *H. filipjevi* genome. (**a**) Hi-C intra-chromosomal contact map of the *H. filipjevi* genome assembly. (**b**) Circos plot of the *H. filipjevi* genome assembly. (1) TRF distribution density; (2) DNA type repeat density; (3) LINE type; (4) SINE type repeat density; (5) LTR repeat density; (6) gene density; (7) GC content.

To acquire a high-quality *H. filipjevi* genome, the draft assembly was further improved using Hi-C analysis with 16.67 Gb Hi-C data. Firstly, the Hi-C reads were mapped to the draft assembly by using BWA^30^. Then, the low-quality reads and duplications were removed to build raw inter/ intra-chromosomal contact maps. Last, based on the agglomerative hierarchical clustering algorithm^31^, Lachesis (Version 201701) was applied for clustering, ordering and orienting, and the scaffolds from genomics were clustered into 9 pseudochromosomes^32^. Finally, Juicebox software (Version 2.20.00) was used to manually correct scaffolded chromosomes and plot heatmap of genomic interactions^33^. Above all, we obtained a 134,189,547 bp *H. filipjevi* genome including 9 pseudo-chromosomes, covering ~89.4% of the whole genome (Fig. 1) and 652 supper-scaffolds, the contig N50 and scaffold N50 are 0.45 Mb and 11.88 Mb, respectively (Table 2). Circos (version 0.64) was used to visualize the *H. filipjevi* genome data^34^.

The completeness of genome assembly was assessed by the following methods. First, the Core Eukaryotic Genes Mapping Approach (CEGMA)^35^ was conducted based on a core gene set involved in 248 evolutionarily conserved genes from six eukaryotic model organisms. The CEGMA evaluation results showed that 248 CEGs assembled 230 genes, with a proportion of 92.74%, indicating that the assembly results were relatively complete. Second, the BUSCO^36^ (version 5.4.3) at genome model was used to evaluate the completeness of genomes in this study using nematoda_odb10 as a database. And we obtained a 55.8% assembly completeness, similar to other reported cyst nematode genomes (43.4–59.3%)^19^. Finally, small fragment library reads were aligned to the assembled genome using BWA software the alignment rate of the total small fragment reads to the genome is about 93.14% and the coverage is about 99.71%, indicating a good consistency between the reads and the assembled genomes (Table 3).

### Genomic repeat annotation

Two technologies were applied to the annotation of repetitive sequences within *H. filipjevi* genome, including homologous comparison and *ab initio* prediction. For homologous comparison, RepeatMasker (Version 3.3.0) and the associated RepeatProteinMask were performed by aligning against Repbase database^37^. For *ab initio* prediction, LTR_FINDER (version 1.0.7)^38^, RepeatScout (Version 1.0.5)^39^ and RepeatModeler (Version 1.0.4) were firstly used for *de novo* candidate database construction of repetitive elements. Followed by, the repetitive sequences were annotated using RepeatMasker, while the tandem repeats were ab initio predicted using TRF (Version 4.07b)^40^. By combining Repbase and *de novo* datasets, we obtained a total of 61.91 Mb of consensus and nonredundant repetitive sequences, which occupied 46.14% of the genome (Table 5).

### Gene prediction and functional annotation

**Fig. 2:**
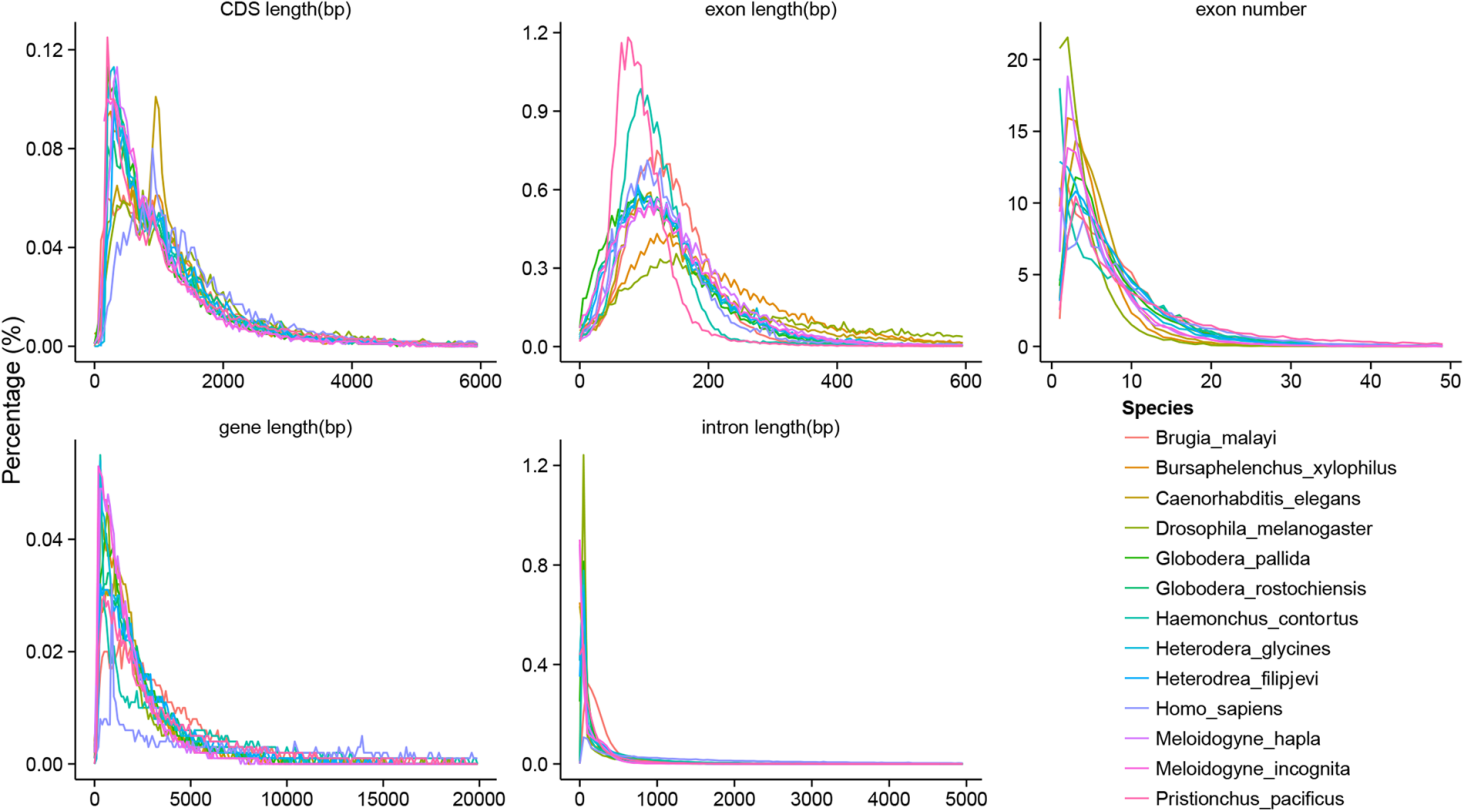
The composition of gene elements in the *H. filipjevi* genome to other species. (**a**) CDS length distribution and comparison with other species. (**b**) Exon length distribution and comparison with other species. (**c**) Exon number distribution and comparison with other species. (**d**) Gene length distribution and comparison with other species. (**e**) Intron length distribution and comparison with other species.

Three approaches were employed for predicting the protein-coding genes within *H. filipjevi* genome, including homology-based prediction, *ab initio* annotation, and transcriptome-based prediction. For homology-based prediction, firstly, protein repertoires of *H. glycines* (GCA_004148225.2)^41^, *G. pallida* (GCA_000724045.1)^42^, *G. rostochiensis* (GCA_900079975.1)^43^, *M. incognita* (GCA_900182535.1)^44^, *M. hapla* (GCA_000172435.1)^45^, *Caenorhabditis elegans* (GCA_000002985.3)^46^, *Haemonchus contortus* (GCA_000442195.1)^47^, *Pristionchus pacificus* (GCA_918442795.1)^48^, *Brugia malayi* (GCA_000002995.5)^49^, *Drosophila melanogaster* (GCA_029775095.1)^50^ and *Homo sapiens* (GCA_024586135.1)^51^ were aligned against the *H. filipjevi* genome using TBLASTN (Version 2.2.29)^52^. Secondly, the BLAST hits were conjoined by Solar software (version 0.9.6)^53^. Thirdly, GeneWise (version 2.2.0)^54^ was used to predict the exact gene structure of the corresponding genomic region on each BLAST hit. Notably, homology predictions were denoted as “Homology-set”. In addition, about 33.2 Gb clean data of RNA-sequencing (RNA-seq) data derived from six developmental stages of *H. filipjevi* were assembled with Trinity (version 2.0)^55^, followed by, the assembled sequences were aligned against *H. filipjevi* genome using Program to Assemble Spliced Alignment (PASA) (version 2.0.2)^56^. The resulting effective alignments were clustered based on genome mapping location and assembled into gene structures. Notably, gene models created by PASA were denoted as PASA-T-set (PASA Trinity set). For *ab initio* annotation, five tools were simultaneously employed, including Augustus (version 3.0.2)^57^, GeneID (version 1.4)^58^, GeneScan (version 1.0)^59^, GlimmerHMM (version 3.0.2)^60^ and SNAP (version 11-29-2013)^61^. Among them, Augustus, SNAP and GlimmerHMM were trained by PASA-T-set gene models. For transcriptome-based prediction, RNA-seq reads were directly mapped to the genome using Tophat (version 2.0.9)^62^. Then the mapped reads were assembled into gene models (Cufflinks-set) by Cufflinks (version 2.1.1)^63^. According to these three approaches, all the gene models were ultimately integrated by Evidence Modeler^64^. The weight of each evidence was set as follows: PASA-T-set > Homology-set > Cufflinks-set > Augustus > GeneID = SNAP = GlimmerHMM = GeneScan. Meanwhile, in order to get the untranslated regions (UTR) and alternative splicing variation information, PASA2 was used to update the gene models. Ultimately, a total of 13,352 protein-coding genes were predicted in the *H. filipjevi* genome. The average transcript length was 3,258.25 bp with an average coding sequence (CDS) length of 1,235.80 bp. The average exon number per gene was 8.14 with an average exon length of 151.89 bp and average intron length of 283.41 bp (Table 6). The statistics of gene models, including lengths of a gene, CDS, intron, and exon in *H. filipjevi* were comparable to those for close-related species (Fig. 2).

In addition, the gene structures of transfer RNAs (tRNA), ribosomal RNAs (rRNA) and other non-coding RNAs in *H. filipjevi* genome were predicted. Specifically, the tRNA were predicted using tRNAscan-SE software (version 1.3.1)^65^. The rRNA fragments were predicted by searching against invertebrate rRNA database using BLAST with an E-value of 1E^−10^. The microRNAs (miRNA) and small nuclear RNAs (snRNA) genes were predicted by INFERNAL (version 1.1.1)^66^ using Rfam database^67^.

**Fig. 3:**
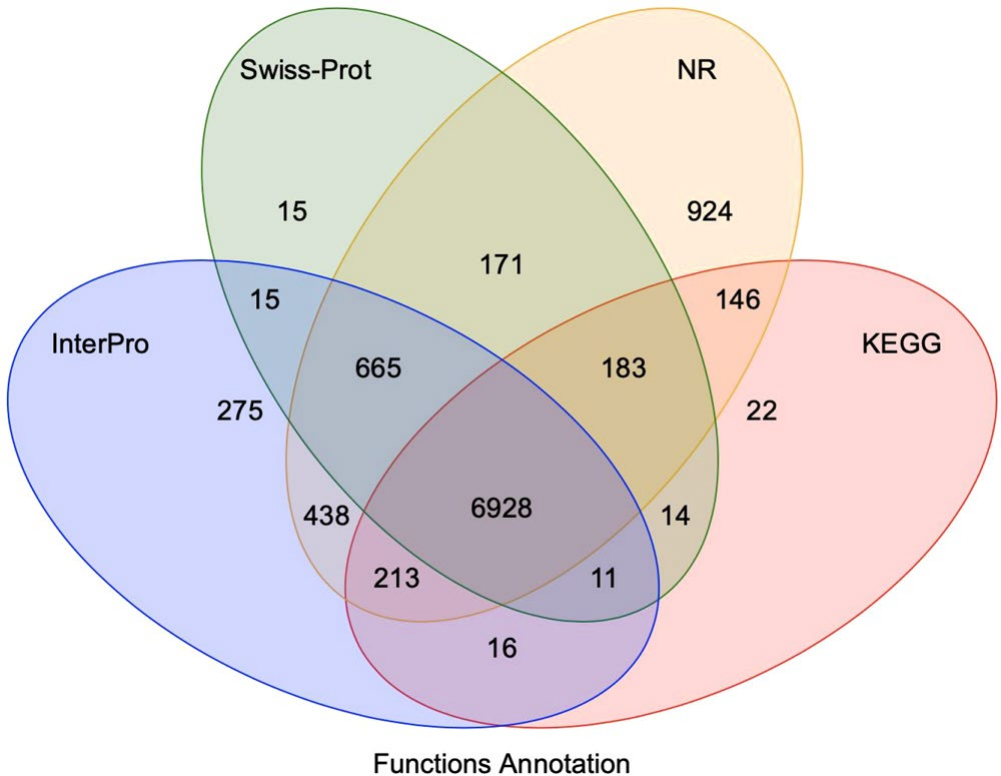
Venn diagram of number of genes with homology or functional classification by each method.

The predicted protein-coding genes in *H. filipjevi* genome were functionally annotated based on homologous searches against databases of SwissProt^68^, NR database (NCBI)^69^, Gene Ontology^70^, InterPro^71^ and KEGG pathway^72^. Notably, InterproScan tool^73^ in coordination with InterPro database^74^ were applied to predict protein function based on the conserved protein domains and functional sites. A total of 10,036 genes (75.20%) were successfully annotated by at least one public database (Fig. 3).

## Data Records

The raw sequence data reported in this paper have been deposited in the Genome Sequence Archive^75^ in National Genomics Data Center (NGDC)^76^, China National Center for Bioinformation/Beijing Institute of Genomics, Chinese Academy of Sciences (GSA: CRA014195)^77^ that are publicly accessible at https://ngdc.cncb.ac.cn/gsa. The genome assembly has been deposited in DDBJ/ENA/GenBank under the accession number JBDPZO000000000^78^, and NGDC under the GSA accession CRA015002^79^. Data of the gene functional annotations had been deposited at Figshare^80^.

## Technical Validation

### Nucleic acid quality

The DNA quality and concentration were assessed using agarose gel electrophoresis and a Qubit 2.0 Fluorometer (Life Technologies, CA, USA).

### Evaluation of genome assembly

Various different strategies were used to evaluate the completeness and accuracy of the *H. filipjevi* genome. First, our assembly genome was verified to have high completeness by CEGMA^35^ (92.74%), indicating that the assembly results are relatively complete. Second, the BUSCO^36^ (v5.4.3) at genome model was used to evaluate the completeness of genomes in this study and other published genome, using nematoda_odb10 as a database. We obtained a 55.8% assembly completeness, similar to other reported plant nematode genomes (43.4–59.3%)^11–14,19^. The low completeness of the BUSCO estimates can be attributed to the substantial genetic divergence between the nematoda_odb10 database and cyst nematodes, with large differences in protein sequences. Moreover, to evaluate the accuracy of the assembly, we used BWA software to align small fragment library reads to the assembled genome to calculate the alignment rate, coverage degree and depth of reads. The results show that the alignment rate of the total small fragment reads to the genome is about 93.14% and the coverage is about 99.71%, indicating a good consistency between the reads and the assembled genomes (Table 3).

## Data Availability

No custom code was used for this study. All data analyses were conducted using published bioinformatics software with default settings, unless otherwise specified.
